# Correction: Novel synthetic route for (parent) phosphetanes, phospholanes, phosphinanes and phosphepanes

**DOI:** 10.1039/d3sc90080k

**Published:** 2023-05-09

**Authors:** Stephan Reichl, Gábor Balázs, Manfred Scheer

**Affiliations:** a Institute of Inorganic Chemistry, University Regensburg Universitätsstraße 31 93053 Regensburg Germany manfred.scheer@chemie.uni-regensburg.de

## Abstract

Correction for ‘Novel synthetic route for (parent) phosphetanes, phospholanes, phosphinanes and phosphepanes’ by Stephan Reichl *et al.*, *Chem. Sci.*, 2023, **14**, 3834–3838, https://doi.org/10.1039/D3SC00580A.

In the above manuscript, we claimed *i.a.* that we reported the first synthesis of the parent phospholane, which was referred to as compound 10b. On closer inspection of the literature, we have realized that our claim is erroneous, since this compound (10b) has been described in two prior papers.^1,2^ We regret overlooking these literature precedents and apologize for our oversight. The synthesis of 10b that we describe is different from those in the two prior reports. The sections of the manuscript that are affected by our mistake are the following. Each entry is followed by a correction or corrected version, in parentheses.

Abstract: “The latter enables the selective synthesis of parent cyclic secondary phosphines (10) in an easy and straightforward way, including the first parent phospholane (10b).” (Corrected version: “The latter enables the selective synthesis of parent cyclic secondary phosphines (10) in an easy and straightforward way.”)

Introduction: The reaction of the spiro compounds 2–5 with nucleophiles leads to the formation of unprecedented heterocyclic parent phosphines 10a–d including the first parent phospholane 10b. (Corrected version: The reaction of the spiro compounds 2–5 with nucleophiles leads to the formation of unprecedented heterocyclic parent phosphines 10a–d.)

Results and discussion: “To our surprise, (parent-)phospholane (HP(C_4_H_8_)) has not been reported so far.” (Correction: This sentence should be entirely omitted from the manuscript.)

Conclusion: In addition to this, we demonstrated that it is also possible to synthesise parent secondary phosphines derivatives 10a–d*via* this route, as represented by the parent-phosphetane HP(C_3_H_6_) (10a) as well as the parent-phospholane HP(C_4_H_8_) (10b), which have been synthesised for the very first time. (Corrected version: “In addition to this, we demonstrated that it is also possible to synthesise parent secondary phosphines derivatives 10a–d*via* this route, as represented by the parent-phosphetane HP(C_3_H_6_) (10a) as well as the parent-phospholane HP(C_4_H_8_) (10b).”)

Finally, we also wish to correct the following sentence “The ^31^P NMR spectra (THF-d_8_) of 10b show a doublet of triplets at *δ* = −70.8 ppm (^1^*J*_P–H_ = 187 Hz, ^2^*J*_P–H_ = 21 Hz)” which contained an erroneous ^1^*J*_P–H_ coupling constant. The correct coupling constant is: ^1^*J*_P–H_ = 180.3 Hz.

The Royal Society of Chemistry apologises for these errors and any consequent inconvenience to authors and readers.

## Supplementary Material
